# What should be the protein target for adjustable Human Milk fortification in premature infants?

**DOI:** 10.12669/pjms.35.1.337

**Published:** 2019

**Authors:** Bayram Ali Dorum, Hilal Ozkan, Salih Cagri Cakir, Nilgun Koksal, Gizem Ezgi Sen

**Affiliations:** 1 Bayram Ali Dorum, Medical Doctor, Division of Neonatology, Department of Pediatrics, Uludag University Medical Faculty, Nilufer–Bursa, Turkey; 2 Hilal Ozkan, Associate Professor, Division of Neonatology, Department of Pediatrics, Uludag University Medical Faculty, Nilufer–Bursa, Turkey; 3 Salih Cagri Cakir, Medical Doctor, Division of Neonatology, Department of Pediatrics, Uludag University Medical Faculty, Nilufer–Bursa, Turkey; 4 Nilgun Koksal, Professor, Division of Neonatology, Department of Pediatrics, Uludag University Medical Faculty, Nilufer–Bursa, Turkey; 5 Gizem Ezgi Sen Medical Doctor, Department of Pediatrics, Uludag University Medical Faculty, Nilufer–Bursa, Turkey

**Keywords:** Adjustable fortification, Human milk fortification, Newborn, Preterm

## Abstract

**Objective:**

To assess the short- and long-term effects of the adjustable fortification (ADJ) regimen on growth parameters in premature infants and to evaluate the amount of protein supplements given to reach the targeted blood urea nitrogen (BUN) levels.

**Methods:**

In this retrospective study, preterm babies who were born at ≤32 weeks gestational age and fed with human milk, were evaluated in two groups. Infants in Group-I were fed only standard fortification (STD). Infants in Group-II were fed the ADJ regimen. The study was conducted between 2011 and 2016.

**Results:**

There were 123 infants in the STD group and 119 in the ADJ group. The mean gestational age of the patients in Group-I was 29.7±1.8 weeks, and mean birth weight was 1266.1±347.1 g. The mean gestational age of the patients in Group-II was 29.5±1.9 weeks, and the mean birth weight was 1217.5±345.5 g. The daily increase in weight and weekly increase in HC were significantly higher in the ADJ group infants. Weight and HC of infants in the ADJ group were significantly higher at 40 weeks. At one year corrected age, weight, length, and HC measurements of both groups were similar. In Group-II, 63% of patients required additional protein supplementation up to 1.6 g/day to achieve the target BUN levels.

**Conclusion:**

A higher protein intake through the ADJ regimen improves the physical growth rate of premature infants in the NICU and after discharge. However, sometimes, the targeted growth and BUN values cannot be achieved despite the administration of protein at the recommended increased doses. Increasing protein supplementation up to 1.6 g/day is safe, feasible, and beneficial for these infants.

## INTRODUCTION

Optimal growth during the care of premature infants in the Neonatal Intensive Care Unit (NICU) is similar to fetal growth.^1^,^2^ However, particularly in very-low-birth-weight (VLBW) infants, postnatal growth restriction continues to be a great problem.^3^ To prevent this, aggressive Parenteral Nutrition (PN) has become a standard practice in NICUs.^4^ When switching to enteral nutrition, human milk is the first and only choice for all premature and term infants. However, the feeding only with breast milk is not sufficient to meet the energy and nutritional requirements of premature infants.^5^,^6^ There is a consensus on the topic of standard fortification (STD) of human milk, particularly for VLBW infants, to ensure the provision of adequate nutrients.^7^

Nevertheless, it was seen that not all VLBW infants achieved the desired growth and brain development with STD.^8^ Thus, individualized fortification methods were developed. ADJ and targeted fortification methods are well-known methods of fortification for preterm neonates.^7^,^9^ These methods have improved growth in VLBW infants have therefore been successfully implemented in NICUs.

The ADJ regimen is being administered to premature infants born at ≤32 weeks gestational age or weighing ≤1500 g in our unit routinely since 2014. However, in some patients, the targeted BUN levels and desirable growth were not reached by the recommended protein supplementation. For these patients, there are no clear recommendations on how to safely give high amounts of protein. In this study, we assessed short- and long-term effects of the ADJ regimen on growth parameters in premature infants born at ≤32 week’s gestation period. In addition, we aimed to elucidate the amount of additional protein needed to achieve the target BUN values in ADJ infants.

## METHODS

This is a retrospective single-center study conducted in a tertiary NICU. In the study, premature babies fed the STD regimen or the ADJ regimen between 2011 and 2016 were examined. Infants, born ≤32 weeks gestational age, who were fed fortified breast milk and whose data in the form of electronic files could be accessed were enrolled to study. Infants with major congenital anomalies, chromosomal anomalies, severe intraventricular hemorrhage, stage 2-3 necrotizing enterocolitis, severe bronchopulmonary dysplasia, and no follow-up were excluded from this study.

Infants fed only STD regimen between 2011 and 2013 were evaluated as Group-I, and those fed the ADJ regimen between 2014 and 2016 were evaluated as Group-II. The prenatal and postnatal characteristics and neonatal morbidities of both groups were recorded.

### Parenteral Nutrition

According to our unit protocol, PN was started in all infants in both groups within the first few hours after birth. On the first day, 3-g/kg/day amino acid solution and 1-g/kg/day lipid infusion were started. After the first day, the amino acid solution was continued as 3.5 g/kg/day. Every day the lipid solution was increased by 1 g/kg/day up to 3 g/kg/day

### Enteral Nutrition

Minimal enteral feeding was initiated as soon as the infant was stable and the first milk of their mothers was obtained. Enteral feeding was increased as 20–30 mL/kg/day. When enteral feeding reached 100 mL/kg/day, PN was discontinued, and 1.1 g of Eoprotin® (Aptamil-Milupa breast milk fortifier) was added to every 25 mL of breast milk until the 40th week. Enteral feeding and energy amounts were increased to 140–160 mL/kg/day and 110–130 kcal/kg/day, respectively, as long as thee infants tolerated the feeding.

### BUN levels and Adjustable Fortification

In Group-II, BUN levels of infants were measured twice weekly. It was aimed to keep BUN levels between 10-16 mg/dL. When the BUN levels were <10 mg/dL, 0.4 g/day protein (Protein Supplement-Milupa, 0.8 g/l g) was added to the infants’ diet including standard fortification. In accordance with BUN levels, the supplemented protein content with 0.4 g/day increments was raised to a maximum of 1.6 g/day. After discharge, infants were evaluated weekly until the 40th week. After the 40th week, the infants were evaluated every two weeks until the 52^nd^ week (12 weeks corrected age). The ADJ regimen was continued until the 52nd week. Human milk protein content was assumed to be 1.5 g/100 mL.

### Growth Characteristics

The weight, length, and head circumference of infants in both groups were documented by the same specialist nurses. Although the length and head circumference (HC) were recorded for infants on a weekly basis, weights were recorded daily. By subtracting birth measurements from discharge measurements and then dividing by the duration of hospitalization, mean daily weight and weekly length and HC growth rates were calculated.

Data from both groups were compared using the Statistical Package for the Social Sciences (SPSS) 23.0 program. Normality test was done with the Shapiro-Wilk test. The t-test was used to compare the means in the two groups, and the chi-square test was used to compare the categorical data. P < 0.05 was considered significant.

## RESULTS

**Fig.1:**
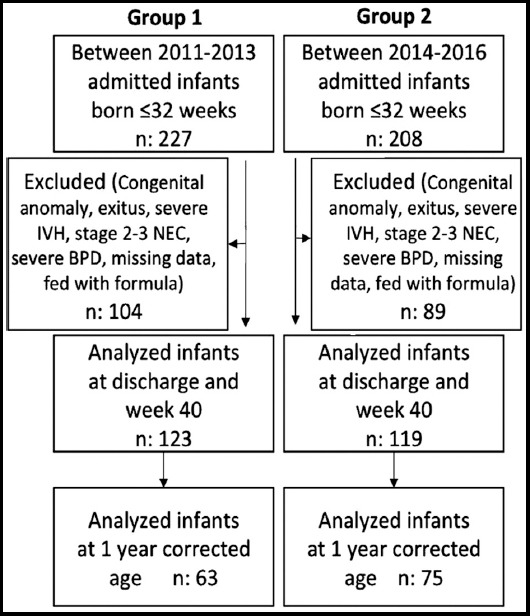
Flow chart of the participant infants.

Numbers of patients included in the study in both periods and excluded from the study are provided in the flowchart (Fig.1). The mean gestational age of the patients in Group-I was 29.7±1.8 weeks, and mean birth weight was 1266.1±347.1 g.

The mean gestational age of the patients in Group-II was 29.5±1.9 weeks, and the mean birth weight was 1217.5±345.5 g. For groups, their demographic and neonatal characteristics, APGAR scores, PN durations, neonatal morbidity rates, and hospital stays were similar (Table-I).

**Table-I:** Demographics and clinical characteristics of infants.

	Group-I (n:123)	Group-II (n:119)	P
Preeclampsia, n (%)	45 (36.5)	41 (34.4)	0.83
Maternal diabetes, n (%)	12 (9.7)	7 (5.8)	0.37
Premature rupture of membranes, n (%)	21 (17)	24 (20.1)	0.65
Male gender, n (%)	66 (53.7)	58 (48.7)	0.52
Intrauterine growth restriction, n (%)	19 (15.4)	13 (10.9)	0.39
Antenatal steroid, n (%)	68 (55.2)	73 (61.3)	0.40
Cesarean section, n (%)	101 (82.1)	94 (78.9)	0.65
Total parenteral nutrition (day) mean ± SD	15 (4-34)	14 (6-39)	0.06
Enteral nutrition started (day), med (min-max)	2 (1-9)	3 (1-8)	0.07
Mechanical ventilation (day), med (min-max)	1 (0-46)	3 (0-42)	0.10
Sepsis, % (n)	40 (32.5)	41 (34.4)	0.85
Respiratory distress syndrome, n (%)	60 (48.7)	57 (47.8)	0.99
Broncho pulmonary dysplasia, n (%)	42 (34.1)	44 (36.9)	0.74
Intraventricular hemorrhage, n (%)	28 (22.7)	28 (23.5)	0.88
Necrotizing enter colitis, n (%)	13 (10.5)	12 (10)	0.90
Hospitalization (day), med (min-max)	40 (15-99)	39 (19-90)	0.99

Although birth weight, length, and HC measurements were comparable for both groups, during their hospitalization, the daily weight gain and HC increase measured weekly in the ADJ group were significantly higher (p <0.05). Similarly, weight and HC of infants in the ADJ group (Group-II) were significantly higher at postnatal week 40 (Table-II). There was no significant difference in the weekly rate of length increase during their admittance and at the 40th week. At the corrected first age, weight, length, and HC measurements of both groups were comparable (Table-II).

**Table-II:** The comparison of the physical parameters all infants.

	STD Group (n:123)	ADJ Group (n:119)	P
Birth HC, cm	27.1±2	26.8±2.2	0.32
Birth weight, g	1266±347	1217±345	0.27
Birth length, cm	37.9±3.6	37.7±3.7	0.76
Increase in HC at NICU, cm/week	0.65±0.2	0.74±0.3	0.01
Weight gain at NICU, g/day	20.4± 5	22.6±7	0.005
Increase in length at NICU, cm/week	0.72±0.2	0.78±0.2	0.07
HC at 40^th^ week, cm	32.5±2.9	33.4±2.8	0.01
Weight at 40^th^ week, g	2794±571	2974±624	0.02
Length at 40^th^ week, cm	47.6±2.7	48.1±2.3	0.12

	*n: 63*	*n: 75*	

HC at 1 year corrected age, cm	44.8±2.1	45.2±1.7	0.18
Weight at 1 year corrected age, g	8396±1044	8533±1212	0.48
Length at 1 year corrected age, cm	73.1±3.1	73.8±4.2	0.30

NICU: Neonatal Intensive Care Unit, HC: Head Circumference.

Sixty-three percent of patients fed the ADJ regimen required additional protein supplementation. Thirteen percent of these patients required protein supplementation at 0.4 g/day, 26% at 0.8 g/day, 12% at 1.2 g/day, and 12% at 1.6 g/day. With this fortification, enteral protein intake in the ADJ group was calculated as 5.1 g/kg/day, while it was 3.9 g/kg/day in the STD group. It was observed that 6% of the infants in the ADJ group could not reach the desired BUN levels with this protein supplementation.

## DISCUSSION

In this study, the short- and long-term effects of the ADJ regimen on the growth of premature infants were examined as compared to premature infants with similar characteristics that were fed only STD. It was observed that infants fed the ADJ regimen have better growth parameters in the short term, consistent with the literature. However, it was observed that babies in both groups had similar growth measures at the one year corrected age.

In the literature, different studies have shown that the ADJ method is easy to apply, is well tolerated by premature infants, and improves their growth without increasing total energy and fluid volume.^10^,^11^ It is stated that the protein amounts recommended for these vulnerable babies could be met with this regimen.^11^ It has also been observed in our study that the growth of infants is better with the ADJ regimen.

The ADJ regimen was first suggested by Moro et al. and was later standardized by Arslanoglu et al.^9^,^12^ This regimen suggests a maximum 1.2 g/day protein supplementation for infants who could not reach the recommended BUN levels (10–16 mg/dL) despite the standard fortification.^13^

However, in our study, 12% of patients did not reach the target BUN values despite protein supplementation of 1.2 g/day. In these patients, protein supplementation amounts were increased up to 1.6 g/day. With this application, the daily protein intake of 3.9 g/kg/day in the STD group was increased to 5.1 g/kg/day in the ADJ group. Nevertheless, it has been seen that not all patients achieved the desired BUN goal or adequate growth. There is no clear recommendation in the literature about the maximum increase in enteral protein intake for such patients.

For a premature infant weighing under 1000 g, ESPGHAN recommends a daily protein intake of 4–4.5 g/kg/day.^14^ Olsen et al. reported that giving high amounts of protein (4.6–5.5 g/kg/day) did not lead to nutritional intolerance or metabolic problems.^15^ Although Cochrane analysis showed that there was insufficient evidence that >4 g/kg/day protein intake was safe, our study found that protein intake of 5.1 g/kg/day was not associated with nutritional intolerance or metabolic disturbance.^16^ Similarly, it has been reported no side effects due to enteral protein uptake at 5 g/kg/day.^17^

In the literature, it was reported that 32% of premature infants weighing under 1250 g required additional protein supplementation.^11^ In our study, this rate was 63% in infants with similar birth weight.

It is important to monitor the premature infants that are well fed during follow-up visits. In a Cochrane analysis, there was no convincing evidence that feeding multinutrient-fortified breast milk after discharge is more advantageous for growth during infancy than feeding breast milk alone.^18^ However, in this study, it was found that infants fed ADJ fortification had better growth at 40th week after discharge.

In the literature, it was reported that infants who were fed individualized enteral protein supplementation had better weight gain, higher increase in head circumference, and increased mental and psychomotor developmental scores at 18 months corrected age.^17^ In fact, in this study, the target BUN levels were between five and nine mg/dL, and these levels were lower than the BUN levels reported by Arslanoglu et al.^13^,^17^ In our study, we did not find any significant difference between the two groups in growth parameters at one year corrected age. Unfortunately, because of the lack of data, the neurological development of the infants could not be evaluated.

### Limitations of the study

It is a retrospective study and the protein content of mother’s milk was not measured. Since it is chronologically later, the improved weight gain of babies in ADJ group may be influenced by the improvements of care in the NICU over the period.

In conclusion, the ADJ regimen improves postnatal growth of premature babies. However, the desirable growth and recommended BUN levels cannot be reached in some patients with the recommended daily protein supplementation quantities. In patients administered the ADJ regimen, in our study shows that increasing protein supplementation up to 1.6 g/day is safe, feasible, and beneficial.

### Authors Contribution

**BAD, HO, SCC, NK, GES:** Conceived, designed and did statistical analysis & editing of manuscript.

**BAD, SCC, GES:** Did data collection and manuscript writing.

**BAD, SCC, HO, NK:** Did review and final approval of manuscript.
